# A novel suprachoroidal microinvasive glaucoma implant: in vivo biocompatibility and biointegration

**DOI:** 10.1186/s42490-020-00045-1

**Published:** 2020-10-14

**Authors:** Ian Grierson, Don Minckler, Marian K. Rippy, Andrew J. Marshall, Nathalie Collignon, Jessica Bianco, Benoit Detry, Murray A. Johnstone

**Affiliations:** 1 Department of Eye and Vision Science, Institute of Ageing and Chronic Disease, University of Liverpool, Liverpool, UK; 2 University of California, Irvine School of Medicine, Irvine, CA USA; 3 Rippy Pathology Solutions, Inc., Woodbury, MN USA; 4 Healionics Corporation, Seattle, Washington USA; 5 Department of Ophthalmology, Centre Hospitalier Universitaire of Liège, Liège, Belgium; 6 NAMSA Clinical and Consulting, Chasse sur Rhone, France; 7 iSTAR Medical SA, Avenue Sabin 6, 1300 Wavre, Belgium; 8 Department of Ophthalmology, University of Washington, Seattle, Washington USA

**Keywords:** Glaucoma, Microinvasive Glaucoma Surgery (MIGS), Suprachoroidal space, Aqueous drainage, Biocompatibility, Biointegration, Eye, Ocular, Ophthalmology, MINIject

## Abstract

**Background:**

A major challenge for any glaucoma implant is their ability to provide long-term intraocular pressure lowering efficacy. The formation of a low-permeability fibrous capsule around the device often leads to obstructed drainage channels, which may impair the drainage function of devices. These foreign body-related limitations point to the need to develop biologically inert biomaterials to improve performance in reaching long-term intraocular pressure reduction. The aim of this study was to evaluate in vivo (in rabbits) the ocular biocompatibility and tissue integration of a novel suprachoroidal microinvasive glaucoma implant, MINIject™ (iSTAR Medical, Wavre, Belgium).

**Results:**

In two rabbit studies, no biocompatibility issue was induced by the suprachoroidal, ab-externo implantation of the MINIject™ device. Clinical evaluation throughout the 6 post-operative months between the sham and test groups were similar, suggesting most reactions were related to the ab-externo surgical technique used for rabbits, rather than the implant material itself. Histological analysis of ocular tissues at post-operative months 1, 3 and 6 revealed that the implant was well-tolerated and induced only minimal fibroplasia and thus minimal encapsulation around the implant. The microporous structure of the device became rapidly colonized by cells, mostly by macrophages through cell migration, which do not, by their nature, impede the flow of aqueous humor through the device. Time-course analysis showed that once established, pore colonization was stable over time. No fibrosis nor dense connective tissue development were observed within any implant at any time point. The presence of pore colonization may be the process by which encapsulation around the implant is minimized, thus preserving the permeability of the surrounding tissues. No degradation nor structural changes of the implant occurred during the course of both studies.

**Conclusions:**

The novel MINIject™ microinvasive glaucoma implant was well-tolerated in ocular tissues of rabbits, with observance of biointegration, and no biocompatibility issues. Minimal fibrous encapsulation and stable cellular pore colonization provided evidence of preserved drainage properties over time, suggesting that the implant may produce a long-term ability to enhance aqueous outflow.

## Background

Glaucoma is composed of a group of linked pathologies, together characterized by the cupping or excavation of the optic disk, degeneration of retinal ganglion cells, and consequently an abnormal visual field [1]. It is a leading cause of irreversible blindness worldwide, affecting more than 64 million people [2]. By 2040, the number of individuals with glaucoma is expected to reach 111.8 million [2]. Elevated intraocular pressure (IOP), although not considered to be a defining feature of the disease, is still recognized as the only risk factor capable of being therapeutically modified [3, 4]. Lowering of IOP is achieved through three modalities: 1) medical therapy, 2) laser intervention, and 3) glaucoma surgery [4]. Glaucoma surgery, with or without a drainage implant, is typically employed when the target IOP cannot be reached with maximal medical therapy or laser treatment.

During the last decade, efforts have focused on the development of “micro-invasive glaucoma surgery” (MIGS) devices to offer patients a less invasive, earlier and safer option for surgical treatment [5]. It is now generally accepted that in all but advanced glaucoma patients, MIGS devices may be considered as an alternative to filtering surgery in the glaucoma treatment paradigm. MIGS devices thus aim to fill the gap between medication and more invasive surgeries [6]. Such devices are intended to promote the drainage of aqueous humor from the anterior chamber towards the physiological conventional (e.g. iStent and iStent inject from Glaukos, Hydrus from Ivantis, Trabectome or Goniotome from MicroSurgical Technology, Kahook Dual Blade from New World Medical, OMNI or VISCO360 or TRAB360 from Sight Sciences, ABiC from Ellex) [4, 7–13] or uveoscleral (e.g. CyPass from Alcon, iStent Supra from Glaukos, MINIject from iSTAR Medical) [7, 14, 15] pathways, or to the non-physiological subconjunctival location (XEN from Allergan, PRESERFLO MicroShunt from Santen) [4, 16, 17].

One major challenge for any glaucoma implant, including MIGS devices, is their ability to provide not only short-term but also long-term IOP lowering efficacy. Notably, the ocular tissues undergo both the normal healing response to the surgical procedure itself as well as a response to the presence of foreign material. The formation of a low-permeability fibrous capsule around the device often leads to obstructed drainage channels and/or an encapsulated filtration bleb. Together, these events may impair the drainage function of devices at both the mid- or long-term intervals [18–23]. These foreign body-related limitations point to the need to develop biologically inert biomaterials to improve performance in reaching long-term IOP reduction.

In the present study, we introduce MINIject^TM^, a novel MIGS implant made of silicone STAR® material. The implant is designed to be implanted in the suprachoroidal space by means of an ab-interno MIGS procedure with retention of a connection to the anterior chamber. The unique structure of the implant consists of an organized porous network of inter-connected spherical voids designed to provide a controlled fluid path for egress of aqueous humor [24]. At the same time, the constituent properties of the device promote biointegration of surrounding tissue into the porous material in a manner that does not induce fibrous encapsulation [25–29]. The purpose of this study is to provide evidence for excellent biocompatibility and tolerability of the MINIject device in ocular tissues, through two different approaches involving in vivo studies conducted in rabbit models. First-in-human results of MINIject at 6 months have since been published [15].

## Methods

Study protocols involving animals were approved by the ethics committee of the contract research organizations which carried out the studies. The biocompatibility study was approved by NAMSA Ethical Committee (NAMSA, France) and was carried out in accordance with OECD Good Laboratory Practice regulations, ENV/MC/CHEM (98)17, European Good Laboratory Practice regulations, 2004/10/EC Directive, and with United States Food and Drug Administration Good Laboratory Practice regulations (21 CFR 58). The time-course biointegration study was designed as a non-Good Laboratory Practice, protocol-controlled study, carried out at the Medanex Clinic (Diest, Belgium), and was approved by Ethical Committee Animal Studies of Medanex Clinic.

### Device description

**Fig. 1:**
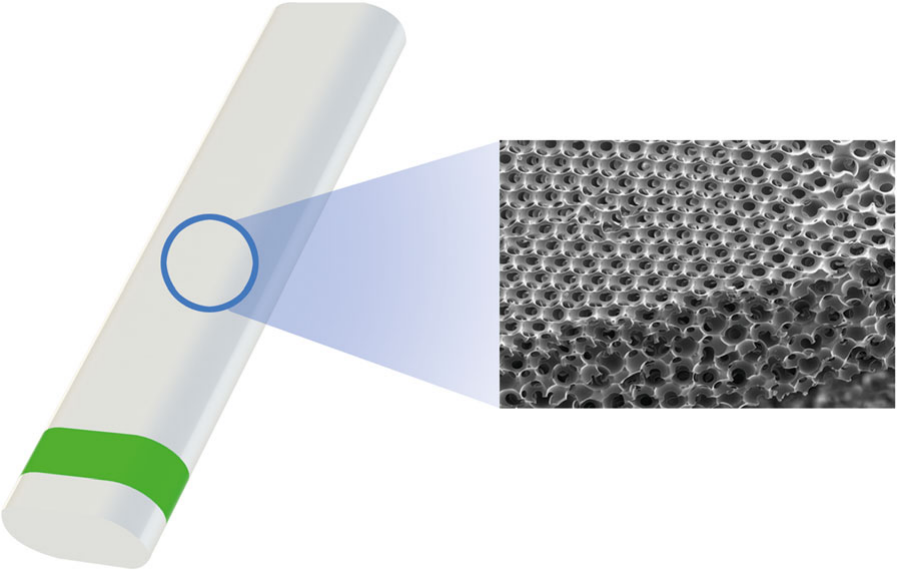
Representation of MINIject (left panel) composed of porous silicone STAR material (right panel)

MINIject is a small oblong glaucoma drainage implant (length = 5.0 mm, width = 1.1 mm, thickness = 0.6 mm), made of a soft, flexible material (Fig. 1). Structurally, the implant is composed of STAR® material, a precision porous construct made of medical grade silicone, i.e. NuSil MED-6215 silicone (NuSil Technology, Carpinteria, CA) designed to maintain long-term stability. The material has a uniform internal pore size of 27 μm. During manufacturing of the material, the sizes of the connections between pores are controlled with high uniformity throughout the entire volume (Fig. 1), to ensure a predictable fluid flow.

The device is intended to be implanted in the suprachoroidal space of the human eye through an ab-interno MIGS procedure under direct gonioscopic visualization with the help of a delivery system. Briefly, the delivery system is introduced into the anterior chamber through a small peripheral corneal incision and the implant is placed in the suprachoroidal space by insertion through the iridocorneal angle. To accomplish this aim, MINIject is designed with a 0.4 mm wide color marking intended to act as a visual clue to control the implantation depth. The marking material is made of long-term medical grade silicone ink, i.e. NuSil MED-6613-6 (NuSil Technology, Carpinteria, CA), chemically crosslinked to the surface of the silicone STAR material.

### Animals and treatments

For both studies, only healthy animals without signs of any significant ocular irritation were selected. Animals were acclimated for a minimum period of 6 days. They were maintained with a 12-h light-dark cycle with free access to food and water. Throughout the in vivo phase of the study, animals were observed daily for any abnormal clinical event. Body weight was recorded weekly.

#### Biocompatibility study

Fourteen (14) female New Zealand White rabbits, with ages of 30 to 33 weeks, were purchased from Charles River Laboratories (Arbresle, France). The animals underwent a MINIject implantation in one eye (Test group) while the contralateral eye either had no operation (Control group), or a sham operation (an identical operation without implant placement, Sham group). Slit-lamp biomicroscopy of the anterior segment was conducted preoperatively, at postoperative days (POD) 1 and 3, and postoperative weeks (POW) 1, 2, 4, 12, and 26. Indirect ophthalmoscopy of the posterior segment was performed preoperatively and on POW 1, 4, 12 and 26. Ocular abnormalities, if any, were recorded preoperatively. At each timepoint after the surgery, all ocular changes were scored following the McDonald-Shadduck scoring system. Intra-ocular pressure was measured diurnally under local anesthesia (1% tetracaine), through tonometry (TONO-PEN VET, Reichert, Depew, NY) preoperatively and weekly after MINIject implantation surgery. Animals were euthanized on POW 12 (*n* = 6) or 26 (*n* = 8), and eyes were enucleated for histopathological analysis.

#### Time-course biointegration study

Twelve (12) female Dutch Belted rabbits, aged 4 to 5 months, were purchased from Covance (Denver, CO). MINIject was implanted in the right eye (Test group) of each animal, while the left eye had no implant nor other operation (Control group). Slit-lamp biomicroscopy was conducted preoperatively, at POW 1, 2, 3 and 4, then at postoperative months 2, 3, 4, 5 and 6 to examine the anterior segment. Intra-ocular pressure was measured diurnally on non-anesthetized corneas through rebound tonometry (TonoVet, Icare Finland Oy, Vantaa, Finland), preoperatively and bi-weekly after surgery. Animals were euthanized on POW 4 (*n* = 3), 12 (*n* = 3) or 26 (*n* = 6), and eyes were enucleated for histopathological analysis.

### Implantation procedure

Similar surgical procedures by qualified glaucoma surgeons were used in both the biocompatibility and biointegration studies using standard aseptic techniques. In the human eye, the MINIject implant is placed through the ab-interno pathway with the help of a specially designed delivery system. However, rabbit eyes have a shallow anterior chamber and narrow iridocorneal angle compared to human eyes, rendering incompatible the use of the delivery system designed for the human eye with safe and optimal implant positioning in the rabbit eye. Thus, substantial changes were made to the surgical approach. These changes involved implant placement through an ab externo surgical procedure as described below.

After induction of general anesthesia, the periocular zone was disinfected, and local anesthetic was applied to the cornea. Each animal was positioned under the operating microscope and an eyelid speculum was utilized. Intrastromal corneal fixation sutures were placed to allow appropriate positioning of the eye during surgery.

A fornix-based conjunctival flap was created in the upper quadrant, followed by the creation of a superficial scleral flap (approximately 50% depth; 3 mm width × 3 mm length). An incision that reached the anterior chamber was made by passage of a blade through the trabecular meshwork. Viscoelastic material (1.2% sodium hyaluronate, Beaver Visitec International, Bidford-on Avon, UK) was injected to fill and maintain a deep anterior chamber during the surgery. Two mm posterior to the incision into the anterior chamber, the remaining layer of deep sclera was incised down to the choroid, reaching the suprachoroidal space. A scleral bridge of 1–2 mm was thus left between the incision into the anterior chamber and the incision into the suprachoroidal space.

Upon careful separation of the sclera from the choroid using an atraumatic spatula, the non-colored posterior portion of the MINIject implant was introduced through the scleral incision and guided posteriorly into the suprachoroidal space. The anterior portion of the device was introduced into the anterior chamber through the trabecular incision so permitting the device to create a connection between the anterior chamber and suprachoroidal space.

Implant positioning was adjusted so that the green mark present on the anterior portion of the implant was half visible in the anterior chamber and the other half remained under the scleral flange. The superficial scleral flap as well as the conjunctival flap were then sutured in a water-tight manner to avoid bleb formation; non-absorbable (Ethilon 9–0, Ethicon, Johnson and Johnson, Diegem, Belgium) and absorbable (Safil 8–0, B. Braun, Melsungen, Germany) sutures were used in the respective tissues. Fixation sutures were removed. The viscoelastic material was removed from the anterior chamber by irrigation with a balanced salt solution (BSS Plus, Alcon, Fort Worth, TX) through a paracentesis incision.

Both test and sham eyes were operated using the same surgical procedure, with the exception that no implant was inserted into the sham eyes; the control eyes remained unoperated. Post-operative medications including a topical drop of anti-inflammatory and antibiotic ophthalmic solution (dexamethasone, neomycin and polymyxin B) 3 times a day, and daily subcutaneous injection of a non-steroidal anti-inflammatory drug (meloxicam) were administered for 7 days.

### Termination and histological processes

After the last examinations were conducted, rabbits were euthanized. In the biocompatibility study, euthanasia of the animals was performed by intravenous injection of 1 mL/kg of pentobarbital (182.20 mg/mL, Dolethal, Vetoquinol, France). Prior to euthanasia, animals were sedated by an intramuscular injection of 10 mg/kg ketamin hydrochloride (Ketamine 1000, Virbac, France) and 2 mg/kg xylazine chlorhydrate (Rompun 2%, Bayer, Germany). In the biointegration study, euthanasia of the animals was performed by intravenous injection of 0.1 mL/kg of T61 (4.39 mg/mL tetracaine chlorhydrate, 26.92 mg/mL mebezonium diiodure, 200.00 mg/mL embutramide, MSD, France). Prior to euthanasia, animals were sedated by an intramuscular injection of 2 mg/kg of xylazine chlorhydrate (Xyl M, V.M.D., Belgium), followed 5 min later by an intramuscular injection of 25 mg/kg ketamin chlorhydrate (Nimatek, Dechra, Netherlands) and 3.5 mg/kg xylazine chlorhydrate (Xyl M, V.M.D., Belgium). Sedation prior to euthanasia was performed for ethical purposes. The eyes were then removed in toto and immersed in appropriate fixative solution for further histopathological processing.

#### Biocompatibility study

Each eye with its proximal optic nerve was macroscopically observed followed by immersion for 24 h in 4% glutaraldehyde mixed 1:1 with 10% neutral buffered formalin. Fixation was then completed in 10% neutral buffered formalin. Eyes of the Test and Sham groups were trimmed along a plane parallel to the surgical site, to provide sagittal sections of the MINIject implant or of the sham-surgical site, respectively. Three sections, separated by approximately 200-μm intervals, were obtained for each eye. For eyes of the Control group, the superior quadrant of the eye was trimmed along a similar plane. The optic nerve was also bisected to obtain longitudinal and transverse sections. Each part of the eye and the optic nerve were dehydrated in a graded series of alcohol, cleared in xylene, and embedded in paraffin. Histological sections were prepared using a microtome followed by staining with Safranin Hematoxylin Eosin. The slides were examined by light microscopy by a qualified pathologist and semi-quantitatively evaluated (based on a scoring system described in ISO 10993-6).

#### Biointegration study

Each eye was macroscopically observed and placed in 10% neutral buffered saline for 3 days. Eyes of the Test group were trimmed along a plane parallel to the implant, with the aim of providing sagittal sections of the device. For eyes of the Control group, the superior quadrant of the eye was trimmed along a similar plane. All samples were processed using a standard paraffin-embedding procedure. Five-μm thick sections were prepared using a microtome for histological and immunohistological investigations. The sections were stained with Weigert’s iron hematoxylin kit (1.15973.0002, Merck, Darmstadt, Germany) and Masson-Goldner Trichrome Staining Kit (1.00485.0001, Merck, Darmstadt, Germany) as per manufacturer recommended instructions.

Immunohistochemical detection of endothelial cells (CD34), lymphatic endothelial cells (LYVE-1), alpha-smooth muscle actin (alpha-SMA), and Type III collagen was achieved with antigen specific target antibodies. Epitope retrieval was performed by heating tissue sections in an epitope retrieval solution (Ultra Cell Conditioning 2, Ventana, Basel, Switzerland) for 20 min at 80 °C in a water bath. The solution was cooled down to room temperature over a period of 20 min to avoid a fast temperature drop and sections were then rinsed twice in distilled water. Endogenous peroxidases were blocked through 15 min incubation in 3% H_2_O_2_ followed by the blockage of nonspecific sites by a 20 min incubation in normal horse serum (ImmPRESS HRP Anti-Goat Ig Polymer Detection Kit, MP-7405, Vector Laboratories, Burlingame, CA), or for 10 min incubation in protein block serum free (X0909, Dako, Heverlee, Belgium), for goat or mouse primary antibodies, respectively.

Sections were further incubated for 1 h at room temperature in polyclonal goat anti-CD34 (1/100; sc-7045, Santa Cruz Biotechnology, Heidelberg, Germany), polyclonal goat anti-LYVE-1 (1/100; AF2089, R&D Systems, Abingdon, UK), monoclonal mouse anti-type III collagen (1/100; NBP2–33328, Novus Biologicals, Cambridge, UK), or monoclonal mouse anti-alpha-SMA (1/500; M0851, Dako, Heverlee, Belgium), followed by 3 washes in phosphate buffered saline (PBS). Sections were then incubated for 30 min at room temperature in ImmPRESS HRP Anti-Goat Ig Polymer Detection Kit (MP-7405, Vector Laboratories, Burlingame, CA) or in anti-mouse EnVision+ System/HRP (K4001, Dako, Heverlee, Belgium), for goat or mouse primary antibodies, respectively. The peroxidase activity was revealed using DAB+ substrate chromogen (Dako, Heverlee, Belgium). Tissues were finally counterstained with Carazzi hematoxylin 0.1%, then dehydrated and mounted with Eukitt medium (VWR, Leuven, Belgium).

Virtual images were acquired with a fully automated digital microscopy system Nanozoomer 2.0-HT (Hamamatsu Photonics, Shizuoka, Japan) using a 40x magnification objective (0.23 μm/pixel) and NDP.scan software (Hamamatsu Photonics, Shizuoka, Japan). Images were visualized using Cytomine software [30]. Histological sections were analyzed independently by four reviewers (IG, DM, MKR and AJM).

### Statistical analysis

Data were expressed as the means ± standard deviations (SD). No statistical analysis of the data was conducted.

## Results

### Biocompatibility study

#### Intraoperative observations

For all operated eyes, i.e. involved in both Sham and Test groups, slight or moderate bleeding from the vascular plexus present in the rabbit sclera was observed during scleral flap dissection. Two eyes of the Sham group and one eye of the Test group displayed a small choroidal prolapse. Consequently, choroidal rupture and extrusion of some vitreous humor were observed for one out of the two sham-operated eyes that had choroidal prolapse. No difficulty was observed during introduction of MINIject in ocular tissues of the Test group. Immediately after surgery, all implants were correctly positioned with the posterior end within the suprachoroidal space and anterior tip visible in the anterior chamber. No implant was damaged during surgery. Cornea or iris damage did not occur in any of the operated eyes. The anterior chambers experienced neither collapse nor the presence of significant blood. The sclera and conjunctiva were successfully closed in all cases.

#### Clinical observations

All animals lost body weight during the first 3 POW, reaching − 5.2 ± 1.7% on POW 2 compared to body weight at surgery. From POW 4, all animals (*n* = 14) started to gain weight and all had recovered at least their initial body weight at POW 12 (+ 8.3 ± 3.5% compared to weight at surgery). At POW 26, all animals (*n* = 8) had gained weight compared to weight at surgery (+ 12.1 ± 5.0%). Signs of ocular or systemic pain and discomfort were absent throughout the study. Together, these data suggest comfort and well-being of the rabbits during the in vivo phase of the study.

In eyes of both the Sham and Test groups, a similar incidence and severity of iris inflammation (graded mild to severe), aqueous flare (graded mild to moderate), fibrin in the anterior chamber (incidence only, severity not graded), cornea cloudiness (graded minimal to mild) and fluorescein staining (graded slight) were observed. Observation of the above findings was present only at early post-operative timepoints and had resolved beyond POW 2. Neither cells in the anterior chamber nor fundus abnormalities were observed in Test or Sham groups throughout the study.

All operated eyes, i.e. sham-operated or implanted with MINIject, showed conjunctival congestion from POD 1 to POW 2 with a similar incidence and severity (mild to severe). Until POW 26, mild conjunctival congestion was present in half of the eyes of the Test and Sham groups. Conjunctival swelling was observed in the eyes of Test and Sham groups from POD 1 to POW 2, occurring with a similar incidence. The severity of conjunctival swelling was slightly higher in the Test group compared to the Sham group on POD 1 but were similar from POD 3 onward (Table 1); findings progressively decreased after POW 2. Similarly, the incidence and severity of conjunctival discharge was slightly increased on POD 1 in the Test group compared to Sham group but were similar from POD 3 onward; the incidence in both groups decreased rapidly after POD 7 (Table 1).

**Table 1:** Slit-Lamp Biomicroscopy conjunctiva examination

	Conjunctival swelling						Conjunctival Discharge					
	POD1		POD3		POD7		POD1		POD3		POD7	
Eye	Test	Sham	Test	Sham	Test	Sham	Test	Sham	Test	Sham	Test	Sham
1	1	1	1	0	1	1	0	0	1	1	0	0
2	1	1	1	1	1	1	1	1	1	1	1	1
3	1	0	1	1	1	1	1	1	1	0	0	0
4	1	1	1	1	1	0	1	0	1	1	0	0
5	1	1	1	1	1	0	1	0.5	1	1	1	0
6	1	0	1	1	1	1	2	0	1	1	0	0
7	2	1	1	1	1	1	1	1	1	1	0	1
8	2		1		1		1		1		0	
9	1		1		0		1		1		0	
10	2		1		0		2		1		1	
11	1		1		1		2		1		1	
12	1		1		1		1		1		0	
13	1		1		1		2		1		1	
14	0		1		1		1		1		0	
Mean	1.14	0.71	1.00	0.86	0.86	0.71	1.21	0.50	1.00	0.86	0.36	0.29
SD	0.53	0.49	0.00	0.38	0.36	0.49	0.58	0.55	0.00	0.38	0.50	0.49

Conjunctival swelling: 0 = normal/none, 1 = minimal, 2 = mild; Conjunctival discharge: 0 = normal/none, 1 = mild, 2 = moderate

Adhesions between the iris and cornea were observed at all time points in the Test and Sham groups, with a progressive increase of the incidence between POD 3 and POW 4. As a consequence of the adhesions, dyscoria was observed at the level of the implant in one eye of the Test group from POW 12. At later time points (POW 12 and 26), a higher incidence was observed in the Sham group compared to the Test group. Endothelial cell density changes were not measured.

Lens opacification was observed in both groups from day 3 onward with the incidence being slightly higher in the Sham group compared to the Test group at POW 12 and 26. Transient cellular deposits were observed in the vitreous in one Test eye from POD 3 to POW 4 while no such deposits were seen in the Sham eyes.

No abnormalities were observed in any eyes of the Control group throughout the study. Overall, slit lamp biomicroscopy and indirect ophthalmoscopy examinations did not provide evidence of differences between eyes of the Test and Sham groups, suggesting that the observations were related to the surgical procedure used for implantation and not due to implantation of the MINIject device itself.

Throughout the in vivo phase of the study, all weekly measured IOP values for Test, Sham and Control groups were assessed to be in the normal range in the New Zealand White rabbit model. No difference in IOP was observed between the groups at any timepoint.

#### Histopathological findings

Following euthanasia on POW 12 (*n* = 6) or 26 (*n* = 8), histological sections including implant (Test group), surgical site (Sham group) or corresponding eye quadrant (Control group), as well as optic nerve, were prepared and analyzed. For the Test group, 1 eye sample of each time point was excluded in the semi-quantitative evaluation since appropriate implant positioning was not confirmed (i.e. both location of anterior portion in anterior chamber and posterior portion in suprachoroidal space).

**Fig. 2:**
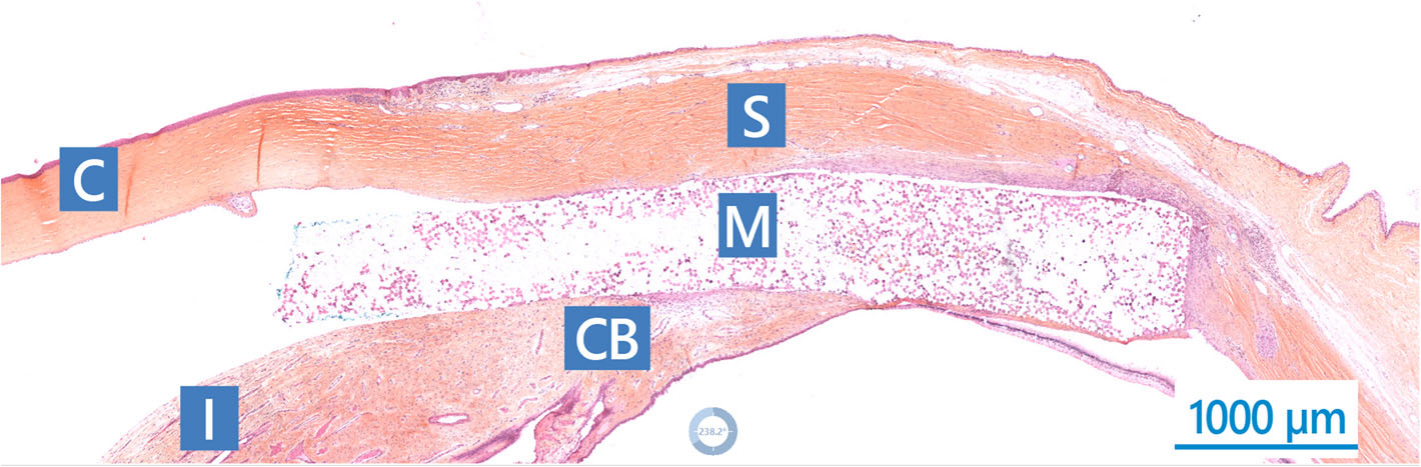
Sagittal section of MINIject in ocular tissues stained with safranin hematoxylin eosin, 12 weeks after implantation in suprachoroidal space of New Zealand White rabbits. C: cornea; I: iris; CB: ciliary body; S: sclera; M: MINIject implant. Scale is 1000µm.

**Fig. 3:**
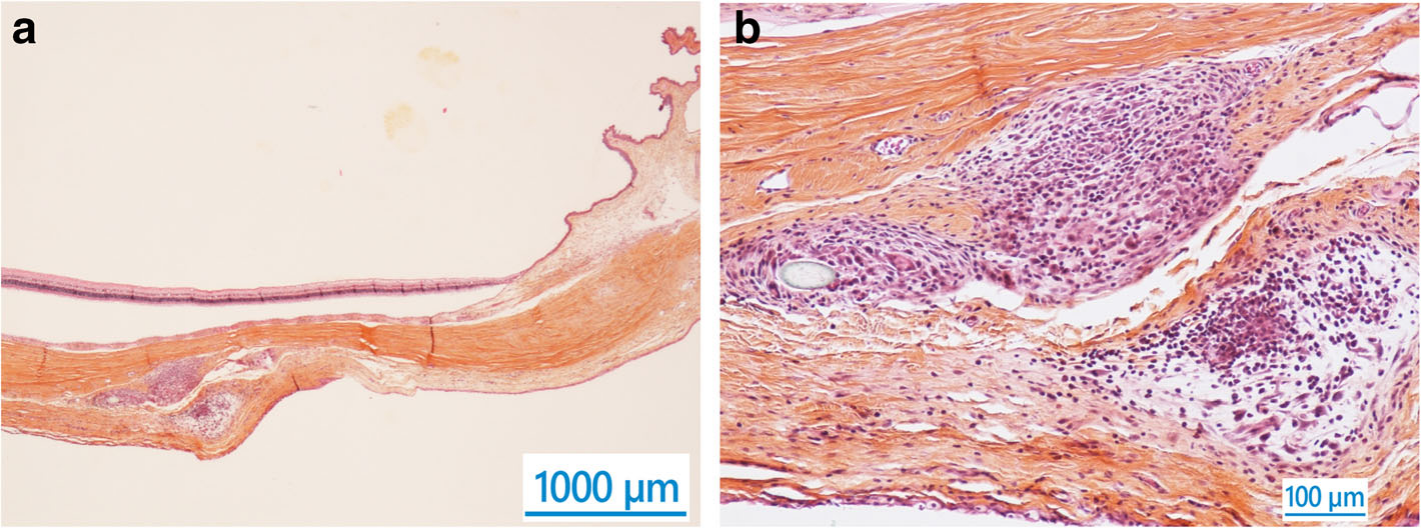
**a** & **b** Accumulation of inflammatory cells in sclera of sham-operated eye (safranin hematoxylin eosin), 12 weeks after surgery. Scale of a: 1000µm. Scale of b: 100µm

In the Test eyes, abundant macrophages and giant cells were observed at the interface between MINIject and host tissues (graded moderate), as well as lower numbers of polymorphonuclear cells (graded slight, Fig. 2). In Sham eyes, these cells were observed in the sclera forming small multifocal aggregates (graded slight) and were in lower amount compared to the eyes of the Test group (Fig. 3). In both Test and Sham groups, lymphocytes and plasma cells were minimal. Only slight fibroplasia surrounded the layer of cells around the implant, providing evidence of absence of implant encapsulation. Fibroplasia was inversely correlated with implant tissue integration (graded moderate to marked). For the test eyes, these observations were similar in terms of both appearance and amount on POW 12 and 26. In the Sham group, a reduced amount of inflammatory cells was present on POW 26 compared to POW 12.

Unique to the eyes of the Test group, a colonization of the porous structure of MINIject was observed (Fig. 2). The colonizing cells were mainly macrophages, but some lymphocytes were also present. Thin strands of newly-formed, vascularized connective tissue were also observed. Tissue ingrowth was graded moderate to marked. The general appearance and amount of colonization was similar at both POW 12 and 26. No sign of implant degradation was observed on POW 12 and 26.

Hemosiderin deposits, estimated as arising from previous surgery-related hemorrhage, were present in the choroid of Test and Sham groups on POW 12. Focal retinal atrophy restricted to the retina lying over the implant was present in 4 of 5 Test eyes on POW 12 and 7 of 7 Test eyes on POW 26 (graded slight to marked). A retinal-targeted biocompatibility issue resulting from MINIject was assessed to be unlikely since the changes were localized to the region of the implant. Because the retinal degeneration was observed in 2 out of 3 Sham-operated eyes on POW 26, although with reduced severity (graded slight), the retinal changes seen were considered to be partly due to the implantation procedure.

No abnormal histopathological findings were observed in any eyes of the Control group. Histological sections of optic nerve of Test, Sham, and Control groups identified no abnormalities, suggesting MINIject had no impact on the optic nerve. No signs of toxic changes related to the MINIject device were observed nor were there any signs of implant degradation.

### Time-course biointegration study

With the aim of investigating MINIject integration in ocular tissues in more detail, an additional study involved suprachoroidal implantation of MINIject in a Dutch Belted rabbit model. Implantation was followed by a time-course histological analysis conducted by 4 independent reviewers. Similar to the clinical observations of the biocompatibility study, no signs of ocular discomfort or systemic pain were observed throughout the study, consistent with the general well-being of the animals. After an initial weight loss, all animals had gained weight upon sacrifice, compared to their weight at surgery (1 month: + 12.9 ± 5.6%; 3 months: + 22.9 ± 8.1%; 6 months: + 38.4 ± 8.8%). Slit-lamp biomicroscopy revealed similar post-operative events in terms of incidence and severity compared to the ones observed in the biocompatibility study. Bi-weekly measured mean IOP values for Test and Control groups were determined to be in the normal range (between 10 mmHg and 20 mmHg) throughout the course of the study. We did not observe any notable difference in IOP values between Test and Control groups.

**Fig. 4:**
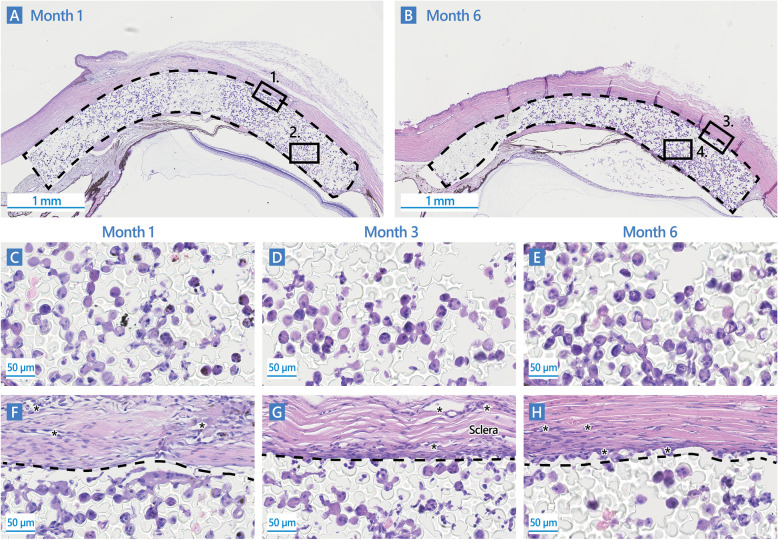
Sagittal section of MINIject in ocular tissues stained with hematoxylin & eosin, 1 month (**a**, **c** and **f)**, 3 months (**d** and **g**), or 6 months (**b, e** and **h**) after implantation in the suprachoroidal space of Dutch Belted rabbits. MINIject is delineated with dotted lines. 1., Magnification in panel **f**; 2., Magnification in panel **c**; 3., Magnification in panel **h**; 4., Magnification in panel **e**; *, neovessels. Scale of 1 mm shown in (**a** and **b**). Scale of 50 μm shown in (**c, d, e, f, g, h)**

Histological analysis was focused on the integration of MINIject subsequent to its implantation in the suprachoroidal location. Overall, tissues directly surrounding MINIject, i.e. sclera and choroid, displayed limited reactivity. A granulomatous reaction was observed in all sections of the Test group, regardless of the post-operative timepoint, and was characterized by an accumulation of cells at the implant’s edges (Fig. 4f-h). The majority of cells were macrophages, but epithelioid cells, giant cells and fibroblasts (also alpha-SMA-positive myofibroblasts), were also present.

Of particular note was the rarity of lymphocytes. A minimal fibrotic encapsulation was present at the interface between the implant and ocular tissues, but deposition of a dense collagen matrix was never observed. Focal neovascularization (anti-CD34-positive neovessels) was observed on most sections in surrounding sclera, ciliary body, and conjunctiva (Fig. 4f-h). All these observations were assessed to be similar, both in terms of incidence and grade, at the different timepoints. Together, these observations suggested that MINIject was very well tolerated in ocular tissues and that the initial tissue reaction did not continue to progress over time.

Similar to the observations made on samples from the biocompatibility study, colonization of the porous implant by cells and extracellular material was present in all histological sections at each of the post-operative timepoints (Fig. 4a-e). Pore colonization density was observed to have a marked gradation. The anterior portion of the implant lying in the anterior chamber had mostly acellular pores while about 50% of pores were assessed to be colonized in the posterior portion of the implant positioned in the suprachoroidal space (Fig. 4a-b). No necrosis, apoptosis, or cell degeneration was observed within the implant, suggesting only healthy cells populated the device in this location.

The most abundant cells present in the pores were of macrophage origin (macrophages and giant cells). Some of the giant cells, extending over several adjacent pores of the silicone material, were characterized by alpha-SMA-positive stress fibers at the level of pore interconnections. In addition, a few fibroblasts were present within the porous structures, rarely expressing alpha-SMA (identifying them as myofibroblasts). However, no fibrosis nor dense connective tissue development were observed within any implant at any time point. The only instance of collagen fibers within pores (including Type III collagen-positive fibers) was in the case of contact between the implant and the cornea. The cornea-implant contact was assumed to be favored by the narrow iridocorneal angle in rabbits (~ 12.5° vs 35–45° in the human eye). Endothelial cell density changes were not measured. No blood nor lymphatic neovessels were in evidence within the porous structures. The pore colonization was deemed to be stable over the time interval between 1 month and 6 months, following suprachoroidal implantation of the MINIject device. No mitosis was observed within the porous structures at any post-operative timepoint. Together, these data suggest that porous structure colonization results from cell migration from surrounding reactive tissues into the porous structure of the implant, and not by any cell proliferation within the implant itself.

## Discussion

Our study provides data on the biocompatibility and tolerability of the novel MINIject MIGS device following implantation in the suprachoroidal space of rabbits. No biocompatibility issue was identified as being induced by the MINIject device after implantation for 6 months into the suprachoroidal space in the rabbit eye. Minimal encapsulation was seen around the implant and was assessed to become stable within 4 weeks after implantation. Implant biointegration, characterized by the colonization of its porous structure by cells of surrounding tissues, was identified and was also assessed to become stable within a short time following implantation (4 POW).

### Biocompatibility

No signs of animals suffering distress were observed throughout both studies, suggesting the device was well-tolerated by the rabbits. Most of the recorded events observed during the period of the post-operative clinical assessments, including mild ocular inflammation, were related to the implantation surgery, rather than being due to the implant itself since the incidence and severity were similar in the Sham and Test groups. Conjunctival inflammation, which tended to be slightly increased in the Test group compared to the Sham group, could be explained by the longer surgery duration. In addition, increased eye manipulation was required for the appropriate insertion of the MINIject, compared to a Sham surgery. Focal retinal atrophy, observed at an early timepoint, occurred with a slightly higher incidence and severity in the Test group compared to the Sham group. However, the retinal changes were not associated with any retinal-targeted biocompatibility issue since retinal changes were not observed at any distance from the implant. The focal retinal atrophy rather appears to result from localized choroidal vascular impairment caused by the physical presence of the implant. Since the surgical positioning of MINIject does not extend over the retina in humans, such a retinal adverse event would not occur and is considered to be an animal model-specific change. No biocompatibility issue was identified following the implantation of MINIject into the suprachoroidal space.

### Clinical evaluation

The rabbits appeared to tolerate the implantation surgery well and they recovered rapidly after the intervention. The majority of post-operative events were thought to be the consequence of the ab-externo implantation surgery procedure, which was the unavoidable consequence of using the rabbit model. In humans, where the use of an ab-interno delivery system is practical, the microinvasive approach is expected to drastically reduce both the incidence and severity of adverse events [31–33]. Notably, the events related to both the conjunctival and scleral incisions should be eliminated and the duration of surgery will be markedly reduced in human patients.

Although we did not observe any clear differences in measured IOP between eyes implanted with MINIject (Test group) and non-implanted eyes (Sham and Control groups), it is possible that unrecognized positioning issues could have affected assessment of drainage efficacy, as appropriate positioning of some devices could not be confirmed. Also, the nature of the normotensive rabbit model complicates the assessment of IOP-lowering performance. Studies specifically designed to assess the IOP-lowering performance of the device are ongoing.

Overall, clinical evaluations of eyes in the Test and Sham groups were similar, suggesting that the observations were related to the surgical procedure used for implantation and not due to implantation of MINIject itself.

### Tolerability and lack of fibrotic reaction

In both studies, histological analysis at 12 and 26 POW revealed minimal fibrous encapsulation and excellent tolerability of the MINIject in ocular tissues of rabbits. The tolerability and lack of fibrous encapsulation are of particular interest because it is well known that rabbits are highly sensitive to ocular injuries and display an aggressive wound healing reaction compared to humans [34–38]. In one historical study, a silicone glaucoma implant produced moderate macrophages and myofibroblasts, with a moderate and continuous capsule surrounding the implant resulting in only 2/6 rabbit eyes patent at 3 and 6 months [39]. In these MINIject studies, tissue reaction to the implant was mostly cellular (granulomatous reaction) with very limited collagen deposition. We assessed that the absence of a dense fibrous capsule surrounding the implant was consistent with the preservation of permeability of the surrounding tissues to passage of aqueous humor. Development of a steady-state of this minimal encapsulation shortly after implantation provides evidence favorable to the maintenance of tissue permeability over time. Evidence of focal neovascularization in surrounding tissues is likely to provide increased extracellular fluid access to the vessels, further promoting the exit of aqueous humor from the eye.

### Biointegration

In association with the minimal fibrosis, biointegration of the MINIject was observed. The biointegration was the result of cells migrating from the surrounding reactive tissue that then entered the porous voids within the implant, a finding in Test eyes of both studies. Biocompatibility and biointegration observations of this study are consistent with those previously reported with STAR® Biomaterial implantation in other sites/tissues [25]. The observed pore colonization into the STAR® Biomaterial of the implant may prevent significant fibrotic encapsulation around the MINIject implant, thus preserving the permeability of the surrounding tissues.

We consider that fluidic properties of MINIject are not impaired by this biointegration process and are preserved over time, since: (1) pore colonization was mainly cellular – cells that are not in sheets containing tight junctions do not block fluid passage, (2) the presence of only healthy cells in the absence of formation of a dense collagen matrix implies that fluid flow through the device is not impeded, (3) pore colonization was only partial - material in the anterior chamber portion of the implant was generally acellular while only ~ 50% of pores were colonized in the most distal portion of the implant in the suprachoroidal space, (4) colonization was stable over time with no evidence of ongoing cell proliferation, (5) thin strands of newly-formed, vascularized connective tissue were observed in the pores which could aid fluid flow from the implant to surrounding tissues, (6) few fibroblasts (or myofibroblasts) were present in the pores, and (7) only nominal collagen fibers were seen.

Together, these data of implant tolerability, a lack of fibrous encapsulation around the implant, and the biointegration of tissues into the pores of the STAR biomaterial support both the presence of long-term tissue permeability and maintenance of drainage performance of the MINIject implant over time.

## Conclusions

The results of these two in vivo pre-clinical studies have demonstrated that MINIject (a novel MIGS device made of porous silicone) is biocompatible and very well-tolerated in ocular tissues when placed into the suprachoroidal space of rabbits. There is very little fibrotic encapsulation surrounding the implant. Biointegration is present and may prevent significant fibrous encapsulation, thus preserving the permeability of the surrounding tissues. Pore colonization, once established, is stable and primarily cellular, supporting the assumption that tissue colonization does not impair fluid drainage through the porous matrix over time. In conclusion, these data strongly suggest that the MINIject implant may provide a long-term ability to enhance aqueous outflow.

## Data Availability

The datasets used and/or analysed during the current study are available from the corresponding author on reasonable request.

## Abbreviations

IOP: Intraocular pressure
MIGS: Microinvasive glaucoma surgery
POD: Post-Operative day
POW: Post-Operative week
